# Developing a sentence level fairness metric using word embeddings

**DOI:** 10.1007/s42803-022-00049-4

**Published:** 2022-10-10

**Authors:** Ahmed Izzidien, Stephen Fitz, Peter Romero, Bao S. Loe, David Stillwell

**Affiliations:** 1 The Psychometrics Centre, Cambridge Judge Business School, The University of Cambridge, Trumpington Street, Cambridge, CB2 1AG UK; 2 Faculty of Science and Technology, Keio University, Tokyo, Japan; 3 Graduate School of Economics, Keio University, Tokyo, Japan

**Keywords:** Text analysis, NLP, Digitisation of human values, Social metrics for texts

## Abstract

Fairness is a principal social value that is observable in civilisations around the world. Yet, a fairness metric for digital texts that describe even a simple social interaction, e.g., ‘The boy hurt the girl’ has not been developed. We address this by employing word embeddings that use factors found in a new social psychology literature review on the topic. We use these factors to build fairness vectors. These vectors are used as sentence level measures, whereby each dimension reflects a fairness component. The approach is employed to approximate human perceptions of fairness. The method leverages a pro-social bias within word embeddings, for which we obtain an F1 = 79.8 on a list of sentences using the Universal Sentence Encoder (USE). A second approach, using principal component analysis (PCA) and machine learning (ML), produces an F1 = 86.2. Repeating these tests using Sentence Bidirectional Encoder Representations from Transformers (SBERT) produces an F1 = 96.9 and F1 = 100 respectively. Improvements using subspace representations are further suggested. By proposing a first-principles approach, the paper contributes to the analysis of digital texts along an ethical dimension.

## Introduction

Given the centrality of texts in the digital humanities, recent work has attempted to leverage word embeddings to reveal information that would otherwise not be readily available. The process of word embedding, as described in this article is based on representing words based on their co-occurrences with other words (Mikolov et al., 2013; Pennington et al., 2014; Rong, 2014). The representation can be considered a digital object that captures meaning (Dobson, 2021). Recent work by Kozlowski et al. (2019), for example, employed word embeddings to separate associations carrying cultural meaning across different time periods, e.g., *rich – poor*, *affluence – poverty.* Word embeddings have also been applied to official state inquiries on social justice (Leavy et al., 2019), as well as on texts related to the COVID-19 epidemic (Aiello et al., 2021). Likewise, Jha et al. (2020) use them to measure popular sentiment towards finance across several decades in which they consider dimensions such as the “*financial system hurts the economy- financial system helps the economy”* to separate between attitudes. While natural language processing (NLP) for textual analysis has been used across several psychological domains such as personality detection (Youyou et al., 2015), a search through the literature for a metric that delivers a fairness score when it is used against sentences produces no records.

In this paper, we ask the question of whether or not it is possible for software to approximate typical fairness perceptions and incorporate them into a measurement tool for simple descriptions of social interactions, one which would allow a sentence describing an interaction between two or more individuals to be classified as *fair* or *unfair.*

Instead of using a philosophical template to define what is *fair* or *unfair*, or by specifying a particular kind of fairness to measure, we approach the problem based on first principles: What are the factors that humans typically use when making a fairness assessment, as found in controlled psychology studies? Is it possible to use these factors in vectors to act as measures of digital texts?

In doing so, we aim to approximate those perceptions, which together form a basis for a *fairness perception*, which we hypothesise will allow sentences to be classified according to which perception they are closer to, being *fair* or *unfair*.

Although the paper does not set out to produce a fully validated and verified fairness measurement tool for documents, it contributes to the development of one based on an approximation of these factors that humans engage when making such measurements. As such, we do not claim to be measuring a specific fairness type, e.g., distributional/outcome. However, fairness evaluations engage several principal psychological factors, which are represented through the use of language – as will be discussed, and it is these factors that we attempt to approximate using a method of word embeddings and vector arithmetic. While the ML techniques used in this paper are well established, our approach to digitising the factors, and the theory behind their use in this domain is new. We are not aware of any such measure that exists in the literature.

On the closely associated topic of morality, several papers have investigated the use of Moral Foundation Theory (MFT) to analyse texts (Graham et al., 2013). The general approach has been to label a dataset of texts with categories and apply an ML algorithm to learn the distinctions between each category (Hoover et al., 2020; Rezapour et al., 2019, 2021). Similarly, Araque et al. (2020) and Pennebaker et al. (2001) use pre-defined measures of moral language. These approaches have proved useful as a form of topic modelling of language, yet they are unable to mark a sentence as being fair or unfair, or offer a degree of explainability as two why a classification was made, and to what degree each sentence is fair or unfair. Indeed a common challenge to such ML systems is the limitations imposed by the technology on explainability (Danilevsky et al., 2020; Dobson, 2020).

Work done by Schramowski et al. (2019) and Jentzsch et al. (2019) have further demonstrated that language models (LM) hold implicit representations of moral values. Their work uses vector comparisons based on a template of Do’s and Don’ts. Furthermore, Schramowski et al. (2019) replicated the moral choices found by Jentzsch et al. (2019), and computed the variance explained by another LM, the Universal Sentence Encoder (USE) (Cer et al., 2018) with respect to Yes-No question templates on moral choices. Further work by Izzidien (2022) replicated the finding that word embeddings contain implicit biases and proposed using them to assess verbs such as ‘thank’ and ‘slur’.

Building on these studies, we propose to harness these implicit moral social biases represented in language to act as a metric for an explainable assessment of sentences without the need for training, specifically those related to fairness. The paper is organised as follows. Section 2 presented next will incorporate a detailed study to determine the most explanatory psychological factors present in fairness assessments. The paper then details two approaches to digitise these psychological factors using word embeddings and ML, one without training, and for comparison, one with training, as will be detailed. We also use two language models, the first being the USE given its use by Schramowski et al. (2019) and Jentzsch et al. (2019). The second being the Sentence Bidirectional Encoder Representations from Transformers (SBERT). We selected SBERT for comparison given its advanced ability to embed contextual information, as will be detailed. The results are subsequently presented. A short discussion is followed by improvements, limitations, and the conclusion.

## Methods

To characterise the factors that humans use when making a fairness assessment, we turn to the psychology literature. Using controlled experiments, social psychologists have considered what factors best explain pro-social acts such as fairness. These studies have involved between-subjects trials and experimental variable manipulations as detailed next.

### The principal factor

One of the challenges of exploring which traits positively predict people acting fairly, is that social interactions often involve feedback between individuals. Thus, asking a participant to share part of a resource with another introduces many confounds, such as social desirability (Platow, 1994), and possible expectations of reciprocity (Fehr & Gächter, 2000). To address this problem, psychologists have attempted to isolate factors that predict prosocial acts by modelling a scenario, in which an actor has the choice to share without any concern for the repercussions of withholding. This has taken the form of the Dictator Game (DG) (Guala & Mittone, 2010).

A DG allows a person to choose, how much of a resource to share with another person, without any concern of being punished, allowing for the removal of strategic intentions (Ibbotson, 2014). A person is typically presented with a pot of cash, which they may keep in its entirety. They may also share part or all of the cash with another player or several players. The researcher often manipulates the context and frame of the study to attempt to decipher which factors influence how much is shared between the players. Spanning 25 years and 20,813 trials, incorporating 24 factors to overcome the limitations of single studies, a meta study by Engel (2011) was conducted on these games. They determined that apart from *age* the strongest positive effects for two person DGs concerned the two variables of recipient *need* and *legitimacy*.

Using the effect sizes (marginal effects) from the meta-analysis as the true population effect size, a second meta-analysis (Ortman & Zhang, 2013) calculated the post-hoc statistical power for the studies included in Engel’s meta-study, which investigate at least one of those explanatory variables by the non-central *t* distribution. They found the effect size for the *deserving recipient* was 1 for four of the studies and above 0.6 for the fifth. *Recipient earned* was under 0.2 and *dictator earned* was close to 0.6 for eleven studies. Apart from a take-option offered to dictators, Zhang & Ortmann (2014) also replicate Engel (2011).

Given that these two factors, *legitimacy* and *need*, were two main psychological contributors to giving, many of the individual studies that found this effect were characterised by their use of the language of *need*, *deservedness* and *entitlement* (Cappelen et al., 2013). A study using the frame: “Note that he relies on you” found that selfish behaviour in the DG almost vanished (Brañas-Garza, 2007). Further, a strong effect for giving was observable that was independent of the extent of altruism measured or of the dictator being seen (Rodrigues et al., 2015). The perception of fairness was demonstrated as being modulated by an integration of the two factors of *egalitarian motivation* and that of *entitlement* (Feng et al., 2013). On entitlement effects, acts of giving were found due to the sense of *earned* shares as evocative of a right that they *deserve* (Cappelen et al., 2010). Such entitlement frames have also been used to explain the observation that individuals in such contexts do not share more of their earned income with those in greater need (Eckel & Grossman, 1996).

It appears that the language encompassing *need* and *entitlement* is evocative of two social values: a right e.g., *he worked for it*, and a responsibility to help: e.g., *he relies on you*, respectively. Both rights and responsibilities may be considered opposite sides to the same coin: If someone has the right to something, then someone else has a responsibility towards that person with respect to that right. As such, responsibility is considered concomitant to a right, as is well established in legal philosophy (Kramer, 2000).

Using 150 observations Tisserand et al. (2015) analysed the two person DGs across seventy papers (1986 to 2014). Their comparative pooled meta-analysis revealed that dictators from countries low in industrialisation exhibited greater considerations for fairness. Industrialisation had a strong negative and significant influence on share. Players from industrialised countries shared significantly less. This was confirmed in Engel (2011) who found that in indigenous countries, a proposer gives more. Such may be reflective of the characteristic of responsibility, which studies report to be influenced by the cultural climate of the person: In a 40 years longitudinal cohort, responsibility was at its lowest when a culture of individualism was at its peak (Helson et al., 2002; Jensen-Campbell et al., 2009; Tisserand et al., 2015). Indeed a study that specifically manipulated the DG to account for mediation effects found that the trait of *social responsibility* was the best predictor of giving (Handgraaf et al., 2008).

Given this principal factor of responsibility, we turned to the wider literature to consider studies that specifically controlled for *responsibility* in their manipulations. These studies were also found to replicate the above finding. A study by van Dijk & Vermunt (2000) asked participants as to the extent they considered it was their responsibility to share the money fairly. They found the unilateral power distribution in the DG triggered a social responsibility norm. The paper found a main effect that those in the DG condition felt more strongly they ought to share their money fairly than those taking part in the Ultimatum Game (UG) setting. Within an UG, a proposer may offer any share of the money to a responder. The responder may accept the proposal, or reject it. If they reject it, neither of them receives any of the money. A study by Yang et al. (2020) found a positive correlation between a sense of community responsibility (SOC-R) and altruism behaviour (AB). Their regressions demonstrated a linear relationship, with SOC-R as the predictor and AB as outcome. In a study by Brañas-Garza et al. (2009) factors of personal involvement and responsibility explained the reasons behind why positive values were given in DGs.

Work by Sijing & Jianhong (2011) used a DG and Third-Party Game to activate the social norm of fairness. They found social responsibility had a critical role in norm activation. After being activated, players who scored higher on responsibility were characterised with greater prosocial behaviour. A study by Milgram (1963) determined that when one was able to make another person responsible for an act, anti-social acts could more easily materialise. Concordantly, Cui et al. (2015) reports that the activations of a person to witnessing others in pain is modulated by the witnessing parties’ responsibility, whereby responsibility sharing, or not being responsible, lowers the pain-matrix neural activity.

One method to attempt to falsify the claim that responsibility plays such a central role, would be to remove or diminish it. A number of studies attempted this manipulation. These are detailed next.

A study by Cryder and Loewenstein (2012) considered whether individuals were more generous in two player DGs than in conditions for which responsibility for any one receiver is potentially divided across more than one dictator. When an individual was completely responsible for somebody else’s outcome, the chances of giving rose by a factor of 3.03. Unambiguous responsibility for a single receiver leads to a higher share. Using a shopping area, a condition was set to elicit a sense of responsibility. Those solely responsible for the outcome of another individual were found to be significantly more generous. In work by Hamman et al. (2010) delegated agents led to settings in which the accountability for questionable moral decisions become diffused, whereby no single person was seen as responsible. Dictators generally preferred to delegate, which led to highly reduced amounts being shared with others.

Bartling and Fischbacher (2012) made an indirect assessment of responsibility. Using a ‘punishment assignment’ for the results of decisions, the measure of responsibility outperformed measures that used inequity aversion or reciprocity to predict punishment behaviour. Lastly, a study by Charness (1998) found that participants responded with more generosity when a random process determined a wage than when assigned by a third party. Such a shift in perceived responsibility for the pay was found to alter behaviour. Participants felt less of an impulse to contribute to an anonymous employer when they perceived that a third party had approved the wage in some way resulting in a shift of some responsibility for the determination of the outcome. They found that individuals are generally more generous with anonymous strangers when they must assume full responsibility for payoff allocation.

### Contingent factors

When a human perceives a context as one that warrants a *responsibility* evaluation (Handgraaf et al., 2008), such evaluation is dependent on contingent factors. By contingent factors, we mean the principal factors needed to allow for a perception of *responsibility* to materialise. Intuitively, these are a perception of the frame (Engel, 2011; Zhang & Ortmann, 2014) in terms of:

1. The benefit-harm gained: A measure of how the actors actions will result in a benefit to the receiver or lack thereof (Brañas-Garza et al., 2014; Bruner & Kopec, 2018; Chiaravutthi, 2019; Perera et al., 2016).
2. The consideration of wider public benefit and harm (Gillet et al., 2009; Lejano & Ingram, 2012; Sigmund et al., 2001).
3. The emotional salience of the context: how much joy-pain is involved (Batson et al., 1991; Edele et al., 2013; Scheres & Sanfey, 2006; Tabibnia & Lieberman, 2007).
4. Outside the DG, a further perception of the possible consequences is incorporated: rewards and punishments (Bartling & Fischbacher, 2012; Boyd et al., 2003; El Mouden et al., 2012, p. 24; Henrich et al., 2001; Nesse, 1990; Scheres & Sanfey, 2006; Strang & Park, 2017).

These principal factors interact in a social context, allowing for a pro-social human propensity, or pro-social bias, to materialise, termed the ultra-cooperative trait, seen as unique to human society (Nowak, 2006; Tomasello, 2014). We will use these factors in word embeddings to act as measures.

### Language and pro-social factors

The use of language has been shown to reflect social perspectives (Kennedy et al., 2021). It has also been shown that a variety of social biases found in the usage of language can be measured when they are used in word embeddings owing to co-occurrences (Pennington et al., 2014), such as demographic features (Kozlowski et al., 2019) and ethnic and gender biases (Garg et al., 2018).

Given these prior findings, a human propensity for pro-social actions, and its articulation in general discourse, may also present in word embeddings, where certain types of social interactions are associated with praiseworthy terms, while others are associated with blameworthy terms, such as *fair* and *unfair* acts respectively. We detail this next.

## Word embeddings as measures

### Approach 1

One of the most pertinent features of word embeddings is their mathematical properties (Pennington et al., 2014). Here words become represented by vectors in an embedding space (Mikolov et al., 2013; Pennington et al., 2014). This vectorisation process includes quantifying word frequencies, probability values, and co-occurrence relations, among other possible options (Dobson, 2021). The linear structure of this resulting embedding space encodes the syntax and semantics of the source language. As such, the vectors can be meaningfully added, subtracted and compared. Comparison can be undertaken using cosine similarity. Closely associated vectors score closer to + 1, with less similar scoring closer to -1, allowing for a measure of how similar vectorised sentences are. The cosine similarity measure is trained using gradient descent to make vectors corresponding to words that appear in similar contexts closer in the embedding space. This is based on the “distributional hypothesis” as expressed in a quotation by the linguist John Rupert Firth - “You shall know a word by the company it keeps!“(Firth, 1958; Cer et al., 2018; Kozlowski et al., 2019).

Given that language reflects the social values of its speakers (Kennedy et al., 2021; Smith, 2010), we hypothesise that word embeddings will reflect the social propensities determined by the psychology literature mentioned above. Thus, sentences that describe fair acts will be more closely associated with sentences that describe responsibility, benefit, joy, and reward, than that of their antithesis terms of irresponsibility, harm, sadness, and punishment. Based on this, it becomes potentially possible to use this feature, this pro-social bias, as a metric. Actions that are typically hurtful will co-occur more with negative social evaluations in typical corpora, reflecting the human propensity towards pro-social acts. As such it may become possible to leverage this bias as a metric.

To use embeddings for this purpose, we propose the method of adding and subtracting vectors (Foley & Kalita, 2016) to narrow the implicit ontological associations of the resulting vector. In using word embeddings, built without any explicit ontological labels, the vector representation of the corpus implicitly reflect ontological knowledge (Bhatia, 2017; Erk, 2012; Racharak, 2021; Runck et al., 2019). For example, grammatical ontologies become reflected due to the co-occurrence of specific grammatical knowledge in the co-occurrence of words (Qian et al., 2016). The term *fairness*, being a collection of several social ontologies may be represented using vectors. This is accomplished using linear combinations of vectors, each of which represent a factor found in the above psychology literature. We use this assumption to ‘triangulate’ a term, i.e., *fairness*, by outlying its main ontologies. In effect, we attempt to incorporate latent vector representations resulting from such addition and subtraction. We detail our method next.

#### The vectors

To represent the psychological factors detailed above as vectors, we constructed the following sentences that describe them (Table 1), which we then converted into vector format using the USE (Cer et al., 2018). Notation wise, a sentence is represented with a lower-case letter, and its vector space embedding by that letter with an arrow on top. For instance, the sentence v = “*it was irresponsible*”, its vector space embedding will be $\overrightarrow{v}$. In cases where no letter is assigned to a sentence, the vector embedding of a sentence is designated by placing an arrow on top of the sentence. For instance, $\overrightarrow{it \ was \ very \ irresponsible}$.

The wording of the sentences were induced from each of the above numbered lists under the *Contingent Factors*. Thus, the two opposite terms of benefit-harm (Brañas-Garza et al., 2014; Bruner & Kopec, 2018; Chiaravutthi, 2019; Perera et al., 2016) were constructed into: $\overrightarrow{^{\prime\prime}it \ was \ beneficial^{\prime\prime}}- \overrightarrow{^{\prime\prime}it \ was \ harmful^{\prime\prime}}$. In considering the wider public benefit-harm (Gillet et al., 2009; Lejano & Ingram, 2012; Sigmund et al., 2001), we constructed: $\overrightarrow{^{\prime\prime}it \ was \ beneficial \ to \ society^{\prime\prime}}- \overrightarrow{^{\prime\prime}it \ was \ not \ beneficial \ to \ society^{\prime\prime}}$. For the emotional salience of the context, i.e., how much joy-pain is involved (Batson et al., 1991; Edele et al., 2013; Scheres & Sanfey, 2006; Tabibnia & Lieberman, 2007), the sentence constructed was $\overrightarrow{^{\prime\prime}it \ was \ joyous^{\prime\prime}}- \overrightarrow{^{\prime\prime}it \ was \ sad^{\prime\prime}}$. Given that outside of a DG, the factors of reward and punishment are contingent factors (Bartling & Fischbacher, 2012; Boyd et al., 2003; El Mouden et al., 2012, p. 24; Henrich et al., 2001; Nesse, 1990; Scheres & Sanfey, 2006; Strang & Park, 2017), the following sentences were used:$\overrightarrow{^{\prime\prime}was \ free \ to \ and \ rewarded^{\prime\prime}}- \overrightarrow{^{\prime\prime}was \ sent \ to \ prison \ and \ punished^{\prime\prime}}.$ As the word ‘free’ can also mean ‘no monetary charge’, we used two opposite terms on each side of the scale to reflect both the material and abstract nature of the consequence, i.e., prison vs. being free (material), and punished vs. rewarded (abstract). Lastly, the principal factor found was framed: $\overrightarrow{^{\prime\prime}it \ was \ very \ responsible^{\prime\prime}}- \overrightarrow{^{\prime\prime}it \ was \ very \ irresponsible^{\prime\prime}}$and given that the quality of ‘responsibility’ was the most pertinent explanatory factor in the psychology studies above – under *the Principal Factor*, this explanatory factor was used with the term ‘very’ to emphasise the range.

Other words which also carry similar meaning may also have been used - as similar words are close to each other in vector space (Erk, 2012) and the cosine similarity result is spatially based and not dictionary based (Han, 2012). How the wordings affect outcome is given in the section on limitations.

The wordings used are given in Table 1.

**Table 1:** Using the principal and contingent factors for vector wordings

Factor	Wording for scale
Responsibility dimension	$\text{it was very responsible - it was very irresponsible}$
Emotional dimension	$\text{it was joyous - it was sad}$
Public benefit dimension	$\text{it was beneficial to society - it was not beneficial to society }$
Personal benefit dimension	$\text{it was beneficial - it was harmful }$
Consequence dimension	$\text{was free to and rewarded - was sent to prison and punished}$

The vectors were constructed:

$$\begin{array}{l}\overrightarrow v^{\left(1\right)}=\overrightarrow{^{\prime\prime}it \ was \ very \ responsible^{\prime\prime}}-\overrightarrow{^{\prime\prime}it \ was \ very \ irresponsible^{\prime\prime}}\\{\overrightarrow{v}}^{\left(2\right)}= \overrightarrow{^{\prime\prime}it \ was \ joyous^{\prime\prime}}- \overrightarrow{^{\prime\prime}it \ was \ sad^{\prime\prime}}\\{\overrightarrow{v}}^{\left(3\right)}= \overrightarrow{^{\prime\prime}it \ was \ beneficial \ to \ society^{\prime\prime}}- \overrightarrow{^{\prime\prime}it \ was \ not \ beneficial \ to \ society^{\prime\prime}}\\{\overrightarrow{v}}^{\left(4\right)}= \overrightarrow{^{\prime\prime}was \ free \ to \ and \ rewarded^{\prime\prime}}- \overrightarrow{^{\prime\prime}was \ sent \ to \ prison \ and \ punished^{\prime\prime}}\\{\overrightarrow{v}}^{\left(5\right)}= \overrightarrow{^{\prime\prime}it \ was \ beneficial^{\prime\prime}}- \overrightarrow{^{\prime\prime}it \ was \ harmful^{\prime\prime}}\end{array}$$

Convex combinations of word vectors can be used to express a semantic gradient ranging between the chosen words. Thus, if we consider the vector describing $\overrightarrow{^{\prime\prime}it \ was \ beneficial^{\prime\prime}}- \overrightarrow{^{\prime\prime}it \ was \ harmful^{\prime\prime}}$, and compare it to a vectorised test sentence, such as $\overrightarrow{^{\prime\prime}the \ guard \ helped \ the \ man^{\prime\prime}}$ through a cosine similarity calculation, the result will be a score from + 1 to -1. The more associated the sentence is with benefit, the closer to 1 will be the result. Whereas sentences that are more associated with harmfulness will provide an outcome closer to -1.

The sentence level *fairness perception* vector $\overrightarrow{v}$ is made by combining the vectors above:

$$\overrightarrow{v}={\overrightarrow{v}}^{\left(1\right)}+{\overrightarrow{v}}^{\left(2\right)}+{\overrightarrow{v}}^{\left(3\right)}+{\overrightarrow{v}}^{\left(4\right)}+{\overrightarrow{v}}^{\left(5\right)}$$

We refer to this as the fairness vector, notwithstanding the limitations described earlier. In using this result, it becomes possible to compare $\overrightarrow{v}$ to the embedding of a test sentence, e.g., “the boy hit the baby” to determine how close the test sentence is in vector space to the parsimonious representations of fairness, by computing the cosine similarity.

In performing the linear manipulation – the addition and subtraction of vectors, the new vector $\overrightarrow{v}$ is able to capture a scale. One that allows for a comparison of a combination of these social dimensions to the sentence being tested.

Were it the case that only one of these social dimensions be used with a test sentence, the result would expectantly not capture the minimum pertinent factors associated with a perception of fairness. To consider this, the results of using each factor ${\overrightarrow{v}}^{\left(1\right)}to\  {\overrightarrow{v}}^{\left(5\right)}$ separately are plot in Section 4 for comparison. The vectors were used against a list of 200 sentences compiled by three independent contributors, aged 24 to 44, all male, and all of professional background.

A test is also conducted to compare the result of using such a parsimonious representation of fairness $\overrightarrow{v}$, against the result obtainable when using the straightforward terms ‘it was fair’ and ‘it was unfair’ for the vector embedding ${\overrightarrow{v}}^{f}$ instead:

$${\overrightarrow{v}}^{f}= \overrightarrow{^{\prime\prime}it \ was \ fair^{\prime\prime}}- \overrightarrow{^{\prime\prime}it \ was \ unfair^{\prime\prime}}$$

It may be that such a vector $({\overrightarrow{v}}^{f})$ will reflect variations on how the term ‘fair’ and ‘unfair’ is used in a corpus. Given the variation of definitions, it would be expected that such a representation would produce conflicting results. This contrasts with building up an ontology of fairness using representations commonly exhibited by humans as determined in the literature review above.

A further three independent human volunteers, aged 21 to 40, two female, one male, and all of professional background, were asked to mark each sentence as either fair or unfair. No further instructions were given to them.

To consider whether the use of the fairness vector $\overrightarrow{v}$ is simply replicating a sentiment analyser, we perform a sentiment analysis. We expect there to be some overlap between positive sentiment and fairness, and between negative sentiment and unfairness, since one may entail the other. However, three issues present themselves on this point. The first being epistemological: The sentence ‘The court convicted the criminal’, for example, is typically considered a description of a fair act. However, the presence of the negatively polarised words ‘convicted’ and ‘criminal’ may lead a sentiment analyser to mark this sentence as negative. Such a result is arguably expected as sentiment analysers are not typically pre-trained on terms that specifically test for the fairness or unfairness of a sentence. To consider this, we test sentences with two main types of sentiment analyser: Dictionary based, e.g., VADER (Hutto, 2021), and language model based, e.g., Robustly Optimized BERT Pretraining Approach (RoBERTa) (Liu et al., 2019).

The second issue relates to pre-trained sentiment analysers whose scores may correlate positively with fairness scores. It is arguable that these analysers may be producing the correct result for the wrong reasons. Essentially exhibiting the black-box problem (Mathews, 2019). This in contrast to using the fairness vector, where we can be aware of the social dimensions being used to test the sentences.

The third issue is that some sentiment analysers use a binary scale, i.e., sentences are classed either as negative or positive, such as feature based sentiment analysers that use Support Vector Machines, for example. Our paper seeks to undertake fine grained analysis by providing a continuous range of scores, thus, we have avoided using such analysers. We have also avoided testing with sentiment analysers which require training in order to discover which labels best represent a fairness scale, e.g., a scale going from the label ‘extremely fair’ to ‘extremely unfair’. This would be the case with an approach that implemented a pre-trained language model for a ‘zero-shot’ analysis for example.

Notwithstanding the above potential limitations of using sentiment analysers we complete the section by testing the 200 sentences using the dictionary based VADER (Hutto, 2021), and language model RoBERTa (Liu et al., 2019) given their ability to produce a range of sentiment scores. The score found for each sentence in each sentiment analyser is then correlated with the scores found when using the fairness vector $\overrightarrow{v}$.

### Approach 2

While adding and subtracting vectors offers a potential method to encompass fairness perceptions into a single vector, some information is inevitably lost by such a reduction. As an alternative we preserve the vectors for each of the evaluations, each as separate dimensions.

As such, we do not perform the above addition of ${\overrightarrow{v}}^{\left(1\right)}+{\overrightarrow{v}}^{\left(2\right)}+{\overrightarrow{v}}^{\left(3\right)}+{\overrightarrow{v}}^{\left(4\right)}+{\overrightarrow{v}}^{\left(5\right)}$, but rather use each independently. Thus, to evaluate a test sentence, e.g., “the shopkeeper assisted the customer”, its word embedding vector $\overrightarrow{s}$ is compared, through cosine similarity, with the each of the five vectors ${\overrightarrow{v}}^{\left(1\right)}$ to ${\overrightarrow{v}}^{\left(5\right)}$, the results of which are stored in a vector ${\overrightarrow{v}}^{m}$. For example, supposing the result of such a cosine similarity operation were:

$$\begin{array}{c}\left(\overrightarrow v^{\left(1\right)},\overrightarrow s\right)=0.2\\\left({\overrightarrow{v}}^{\left(2\right)},\overrightarrow{s}\right)=0.1\\\left({\overrightarrow{v}}^{\left(3\right)},\overrightarrow{s}\right)=0.6\\\left({\overrightarrow{v}}^{\left(4\right)},\overrightarrow{s}\right)=0.3\\\left({\overrightarrow{v}}^{\left(5\right)},\overrightarrow{s}\right)=0.2\end{array}$$

The stored result ${\overrightarrow{v}}^{m}=\left[0.2, 0.1, 0.6, 0.3, 0.2\right]$

This is repeated for all test sentences, resulting in a dataset D1, which is then hand labelled with the correct fairness assessment (Table 2). This produces a dataset containing the vector and its label.

**Table 2:** Snippet of dataset D1

Index	Test sentence	Result${\overrightarrow{v}}^{m}$	Label
1	*the shopkeeper assisted the customer*	$\left[\text{0.2,0.1,0.6,0.3,0.2}\right]$	Fair
…			…
200	*the prisoner murdered the inmate*	$\left[-0.4,-0.6,-0.3,-0.3,-0.4\right]$	Unfair

To use the Dataset D1 for training a classifier, we perform ML using a logistic regression classifier, and a 1:7 test split. To encode the sentences, we used the USE (Cer et al., 2018), detailed next.

To compare the results of using the USE against another language model, we employ Sentence Bidirectional Encoder Representations from Transformers (SBERT), which is also detailed next. We encode the sentence level *fairness perception* vector $\overrightarrow{v}$ using SBERT and re-test the list of sentences given in Appendix 2, which are also encoded by SBERT.

To explore how the factors induced from the psychology literature explain the data, a principal component analysis (PCA) is performed with two components. In PCA, the first principal component accounts for most of the variance in the data. Whereas the second component accounts for the second largest amount of variance in the data and is uncorrelated with the first principal component.

#### The universal sentence encoder

Initially, shallow pre-training of early model layers became standard in NLP research through methods such as Word2vec (Mikolov et al., 2013). Subsequent progress followed trends similar to those in computer vision, which naturally led to pre-training of multiple layers of abstraction. These advancements resulted in progressively deeper hierarchical language representations, such as those derived using self-attention mechanisms in transformer-based architectures (Vaswani et al., 2017). Current state-of-the-art NLP systems use representations derived from pre-training of entire language models on large quantities of raw text, and often involve billions of parameters. The success of neural network-based ML models, especially those involving very deep architectures, can be attributed to their ability to derive informative embeddings of raw data into submanifolds of real vector spaces. The common idea behind these developments is that we can learn syntax and semantics of natural languages by training a Deep Learning (DL) model in a self-supervised fashion on a corpus of raw text. Modern embedding methods combine word and sub-word (e.g., morpheme or character) level embeddings in a hierarchical and contextualised fashion to produce sentence and document level representations into (usually high-dimensional) submanifolds of R^*n*^.

Given the high costs and low availability of manually labelled texts for training NLP models, word transfer models deploy pre-trained word embeddings (Mikolov et al., 2013; Pennington et al., 2014), which were successfully adapted to sentence-level representations (Conneau et al., 2017), and in particular utilised within the encoding module of the USE.

The USE architecture can be deployed with a variety of embedding modules. The options mentioned by the authors included transformer sentence encoding providing high accuracy (which was not used in initial experiments but mentioned as an alternative), and one deploying a deep averaging network (DAN), which focuses on computational efficiency.

This combination of features presented by the USE made it a good choice for our work. First, the simple DAN module employed in the USE encoder makes it somewhat a compromise between predictive power and computational efficiency. Second, it marks a midpoint between sparser and more explainable models and deeper black-box architectures. This trade-off between explainability and accuracy is especially useful in the context of our work. Shallow models are closer to typical statistical learning and analysis procedures, which are prevalent in psychology and computational social sciences today, which make them ideal to study the ramifications of defining model components based on psychological theory.

Since the transformer side of the USE allows us to derive powerful context sensitive representations for natural language inputs, while on the other hand, the DAN side of USE allows us to inject these ethical considerations into the final representations of sentences produced by the combined encoder modules, it is particularly useful for work combining theory driven ethical considerations with natural language modelling methods. Our choice of USE allows us to impose knowledge derived from psychological findings. Such would be hard to do in a fully unsupervised setting. This has the further benefit of combining transparency and efficiency.

On a technical level, the USE first transforms languages to lower-case and tokenises them via the PennTreebank (PTB) (Taylor et al., 2003). In both variants, a 512-dimensional embedding is produced. The transformer encoder deploys sub-graph encoding (Vaswani et al., 2017) to create sentence embeddings through a six-layered stack, whereby at each layer, a self-attention mechanism is followed by a feed-forward network. Words are fed through these layers, and their order as well as their context is taken into account through the use of positional embedding and sentence level attention mechanism. This process iteratively enriches the representation of each word in order to augment the resulting embedding with contextual information of the sentence in which it appears within the corpus.

Each embedding is then added together, whereby the length difference of sentences is ‘standardised’ by dividing through the square root of the length. This results in an output sentence embedding in shape of a 512-dimensional vector, which is then fed into downstream tasks. The DAN variant is based on deep averaging networks (Iyyer et al., 2015) and follows a simpler approach, which starts by averaging embeddings for both bi-grams and words, and then passing these through a four-layered neural network output module.

To ensure general purpose deployability, the transformer encoding uses multi-task learning, whereby one input model is fed into several downstream tasks. First, unsupervised learning is achieved through a Skip-Thought resembling task, replacing the encoder by the above two variants of input models (Kiros et al., 2015). Second, the input-response task for parsed conversational data, which deploys the same encoder for input and output to model the difference of both, whereby their dot product determines the respective relevance, is fed through a softmax function, resulting in an optimisation over log likelihood of obtaining the correct response (Henderson, 2017).

Last is the classification task using sentence pairs that represented the premises, hypotheses, and judgements about each pair. In this task, encoder outputs are processed by fully connected layers and a three-way softmax, resulting in the probability of a judgement for each pair, which resembles earlier approaches (Conneau et al., 2017) to the task of natural language inference.

Finally, for classification transfer tasks, the respective outputs are fed into a specific deep neural network, whereas for the pairwise similarity task, the similarity is calculated in the following way:

First, the cosine similarity of two sentence embeddings is computed, then, the angular distance is obtained by applying the *arccos* function (Eq. 1) to the normalised inner product of the corresponding sentence representations.

1 $$sim\left(\overrightarrow{u}.\overrightarrow{v}\right)= \left(\frac{1-arccos\left(\frac{\overrightarrow{u} . \overrightarrow{v}}{\parallel \overrightarrow{u}\parallel \parallel\overrightarrow{v} \parallel}\right)}{\pi }\right)$$

#### Sentence bidirectional encoder representations from transformers

One of the breakthroughs in deep neural language models came from a novel use of attention in neural networks. Attention was initially introduced as an improvement to recurrent encoder-decoder architectures (Bahdanau et al., 2016) in the context of neural machine translation systems. Subsequently, it was discovered that the idea of attention alone can be used as a basis for language modelling systems. A seminal paper under the title “Attention Is All You Need” (Vaswani et al., 2017) introduced a new type of neural network architecture for extracting deep contextualised text representations from raw natural language data using a process based predominantly on repeated application of the “self-attention” operation in a model called the transformer. This kind of model transforms original vector space representation of linguistic units through a sequence of embedding spaces, where each successive mapping recomputes the representation of every token in the context of its surrounding tokens. This allows for the semantics of words as seen by the neural artificial intelligence systems to vary depending on the context and evolve over time. Such representations produce significant performance improvements on natural language understanding tasks.

Attention based encoders are usually implemented in the context of autoregressive language modelling. The fundamental goal of language modelling is to assign high probability to utterances (usually sentences in plain text) that are likely to appear in data (i.e. belong to the language) and low probability to strings of words that are not. A trained language model can then be used to assign probability to arbitrary sequences of words.

In the past, language models were parametric statistical models estimated from data. However, they have since been replaced with much more successful deep neural network based approaches. Generally, a neural language model is a neural network taking strings of words as input, and returning a scalar probability of the those strings, which is trained to correspond to the likelihood that such a string conforms to a particular language, as induced from large quantities of text, often called a corpus:

2 $$\mathbb{P}(s)=\mathrm{DNN}\;\left(\alpha\left(s\right)\right)$$

where *s* = ${\omega }_{1}{\omega }_{2}\dots {\omega }_{n}$ is a string of linguistic tokens (usually words), and *α* is some input embedding (usually into a distributed vector space representation induced by a neural network encoder).

Normally, instead of thinking of a language model in terms of estimating joint probability of a string of words, we view it in terms of its ability to predict continuation of an input sequence. To obtain this interpretation, we apply the chain rule to decompose the joint probability of a string of words into conditional probabilities of generating a word following a sequence of words already generated:

3 $$=\mathbb{P}\left(\omega_1,\omega_2,\dots{,\omega}_n\right)=\mathbb{P}\left(\omega_1\right) \mathbb{P}\left(\omega_2|\omega_1\right) \mathbb{P}\left(\omega_3|\omega_1, \omega_2\right) \dots \mathbb{P}\left(\omega_n|\omega_1, \omega_2, \dots, \omega_{n-1}\right)$$

A neural language model therefore focuses on computing a conditional probability of word ${\omega }_{n}$ following a sequence of words ${\omega }_{1},{\omega }_{2},\dots ,{\omega }_{n-1}$.

The most successful recent approaches to language modelling are based on the idea of self-attention. The predominant neural architecture for linguistic unit representations based on it, is called the transformer. It is similar to the attention in encoder-decoder models for sequence mapping, except it can be done inside the encoder, directly on the input representations. The goal is to transform the initial input representation through a series of re-representation steps, where the embedding of each token is recomputed as a mixture of embeddings from its surrounding tokens. If the tokens are word vectors in a sentence, we can understand this as generating “contextualised” word representations. In this case, and in contrast to shallow linguistic unit representations such us word2vec (Mikolov et al., 2013), the word vectors are not constant but evolve over a number of steps, where each word vector is re-expressed based on its context in the particular sentence being processed.

At the time of writing of this paper, all top performing language models are deep transformer based architectures. Because of this, in addition to USE embeddings, we perform our analysis with sentence embedding vectors based on BERT (Devlin et al., 2019).

BERT is a transformer language model that was pretrained on large quantities of text using masked sequence prediction, as well as a next sentence prediction task (where the goal is to maximise probability of consecutive sentences found in the training corpus). Token embeddings obtained from BERT yield very informative general purpose features used in many downstream NLP tasks. It is tempting to use these token representations as a basis for a naive sentence embedding, by computing their average in a fashion similar to the DAN implementation of the USE model. However, such naive sentence representations give rather disappointing results when used in standard NLP tasks (even much simpler methods such as Glove (Pennington et al., 2014) perform better with this approach). An early approach to producing useful sentence embedding with BERT was by use of a cross-encoder to train a similarity measure over pairs of sentences. However, this solution was not scalable to real world data sets due to high computational footprint of performing a full forward inference through a BERT model for every pair of sentences. An efficient solution to producing sentence embeddings with BERT involves the use of a siamese network architecture (Reimers & Gurevych, 2019). Sentences are processed through BERT as usual, and the outputs from the final self-attention layer are collapsed into a single vector by use of pooling and projection layers stacked on top of the encoder. In order to fine tune these embeddings, the most prominent method is the soft-max loss approach. The training using this approach is usually done on a natural language inference task such as Stanford Natural Language Inference Corpus (Bowman et al., 2015) and the Multi-genre Natural Language Inference Corpus (Nangia et al., 2017). These tasks involve predicting one of three possible classes for a pair of encoded sentences: entailment, neutrality, contradiction. First we encode two sentences, by passing them through BERT layers and pooling, which produces two vectors u and v. The standard approach to fine tune the encoder using the NLI task is then to compute a vector concatenation of the form $\left[u,v,\left|u-\left.v\right| \right.\right]$. This combined representation is then fed into a Feed Forward Neural Network (FFNN) producing three activations, which are then trained using the softmax approach to compare them with the labels found in the NLI dataset. There exist various optimisations to this basic approach such as multiple negatives ranking, which produce improved performance for various types of natural language processing problems. Finally, we note that there have been many developments on the topic of sentence embedding from transformer language models since the original SBERT paper. These include models such as RoBERTa (Liu et al., 2019) and MPNet (Song et al., 2020).

## Results

The three independent human volunteers marked the 200 sentences appearing in Appendix 2 as fair or unfair. Their designations all matched the original sentence categories: 100 fair sentences and 100 unfair, as appears in Appendix 2.

### Approach 1

We begin by considering 36 sentences (8) selected randomly for graphical illustration purposes from the list of 200 sentences. Each test sentence is compared through a dot product with the vector:

$${\overrightarrow{v}}^{f}= \overrightarrow{^{\prime\prime}it \ was \ fair^{\prime\prime}}- \overrightarrow{^{\prime\prime}it \ was \ unfair^{\prime\prime}}$$

**Fig. 1:**
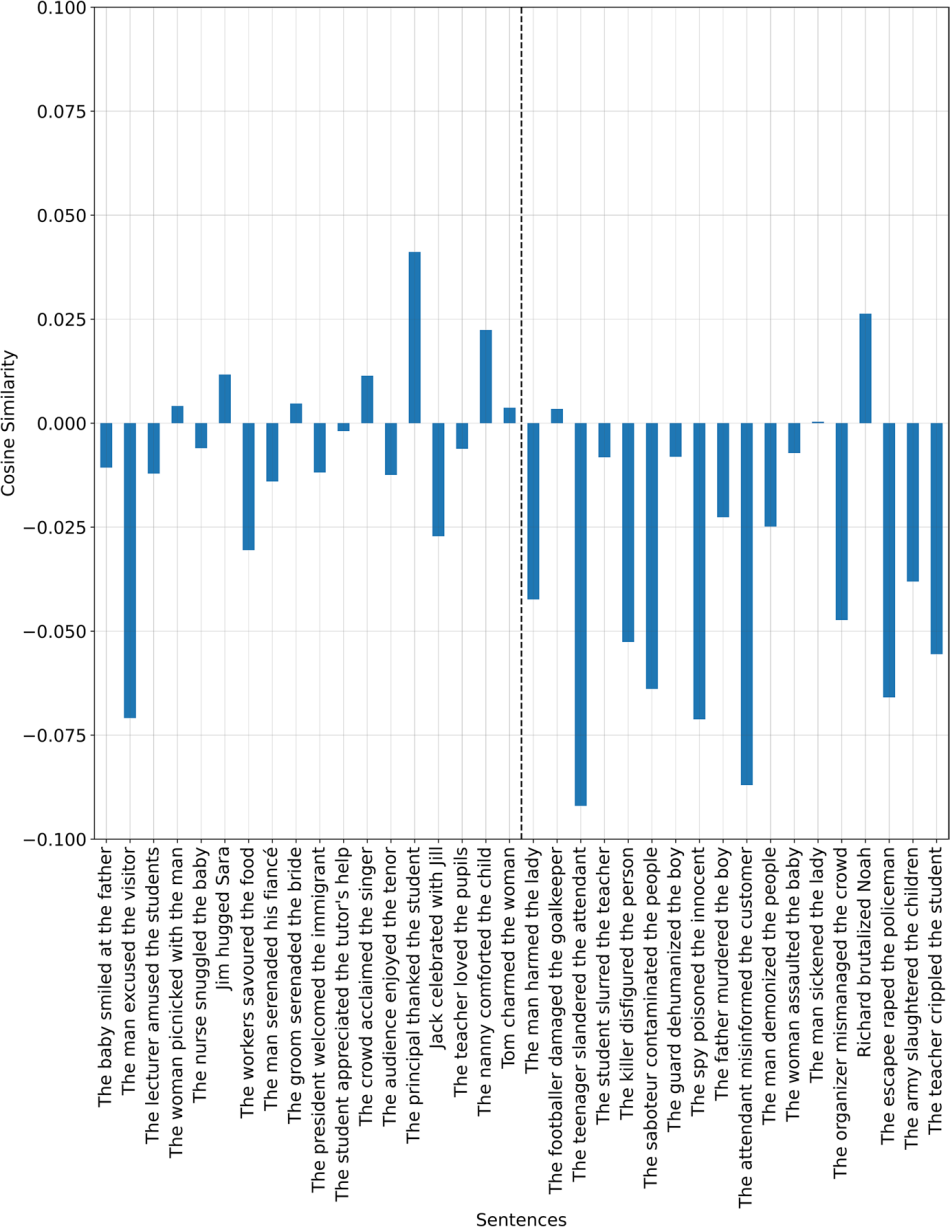
Using ${\overrightarrow{v}}^{f}$results in incorrectly classified sentences. All sentences of the left of the dotted line ought to be positive, while all sentences on the right ought to be negative. The incongruence of the scoring of the unfair sentences on the right can also be seen by comparing the score for *murder (-0.024)* to that of the act of *misinforming (-0.087)*

This produces an incorrect result. Fourteen of the sentences are misclassified (Fig. 1). Correctly classified unfair sentences are classified in a manner that does not necessarily reflect typical evaluations: ‘The teenager slandered the attendant’ is classified as closer to *‘it was unfair’* than *‘the father murdered the boy’* by orders of magnitude. Furthermore, it can be seen that the false negatives are comparatively high, as given on the left part of the figure, but at the same time this is also true of the true negatives, as given on the right part of the figure. We hypothesise that this result may be due to second order associations made with the fair sentences. Whereby they may co-occur more regularly with unfair terms, for example the phrase which received the lowest score: ‘the man excused the visitor’, may be associated with an unfair act mentioned in the training corpus, one that frequently occurs with the term ‘excused’. This is likely because fair acts typically need not be excused, only unfair acts. This theory is also augmented by the magnitude being comparable to the true negative scores on the right hand side of the figure, in which the outcomes can be seen to reflect associations with acts of unfairness.

**Fig. 2:**
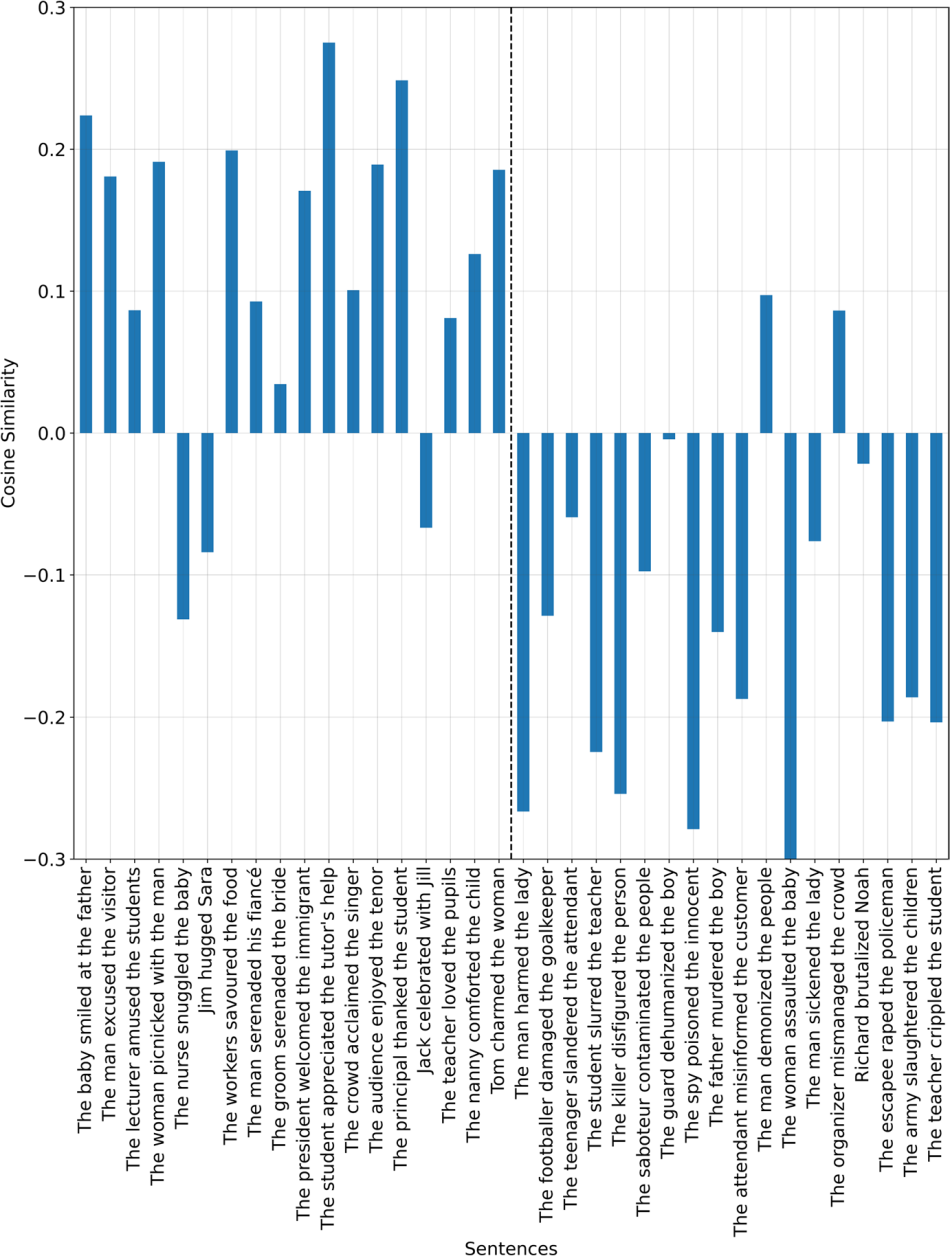
Use of the fairness vector $\overrightarrow{v}$ to measure the similarity of each sentence with a parsimonious representation of fairness

If the fairness vector $\overrightarrow{v}$ is used instead, the results are shown in Fig. 2.

The results seen in Fig. 2 may be said to be a closer representation of typical fairness evaluations when compared to the results seen in Fig. 1.

**Fig. 3:**
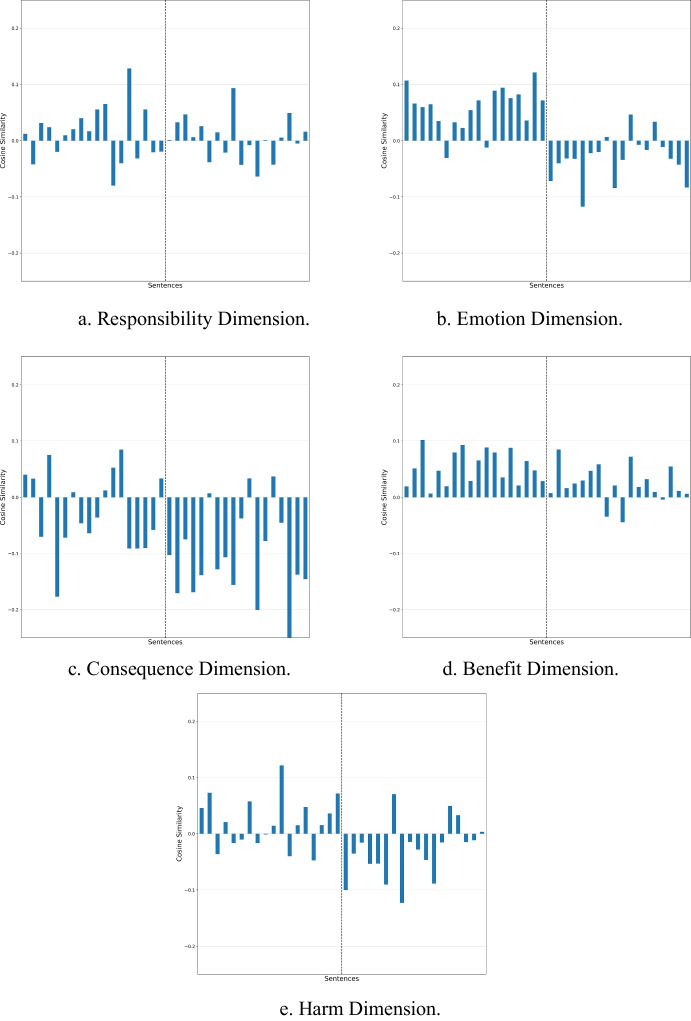
Outcome of using each of the five ranges represented in (${\overrightarrow{v}}^{\left(1\right)}to\  {\overrightarrow{v}}^{\left(5\right)}$ ) with the illustrative 36 sentences in Appendix Table 8. Bars on the left of the dotted line ought to be positive while bars on the right ought to be negative. Each figure represents a dimension of a fairness perception, and thus captures partial information regarding how associated each sentence is with fairness/unfairness. **a** Responsibility Dimension. **b** Emotion Dimension. **c** Consequence Dimension. **d** Benefit Dimension. **e** Harm Dimension

As a means to demonstrate how each of the ontologies gives separate results when they are used singly, Fig. 3 is plot. This represents the outcome of using each of the five vectors: ${\overrightarrow{v}}^{\left(1\right)}to {\overrightarrow{v}}^{\left(5\right)}$ independently. Thus, for example, Fig. 3a reflects how similar each sentence is with the phrase: *“it was very responsible” - “it was very irresponsible”. *Eleven unfair sentences are misclassified as responsible. Each cosine similarity outcome is plot for each vector range as given in Fig. 3a and e. Here we observe how each sentence score is a reflection of factors contained within the corpus. For example, most of the test sentences describing fair acts, are classified as having a negative consequence (Fig. 3c).

Adding and subtracting them produces the aforementioned fairness vector, $\overrightarrow{v}=$ ${\overrightarrow{v}}^{\left(1\right)}+{\overrightarrow{v}}^{\left(2\right)}+{\overrightarrow{v}}^{\left(3\right)}+{\overrightarrow{v}}^{\left(4\right)}+{\overrightarrow{v}}^{\left(5\right)}$ and produces Fig. 2 for the same sentences. For which a more typical reflection of fairness perceptions is obtainable, though not perfectly accurate.

**Fig. 4:**
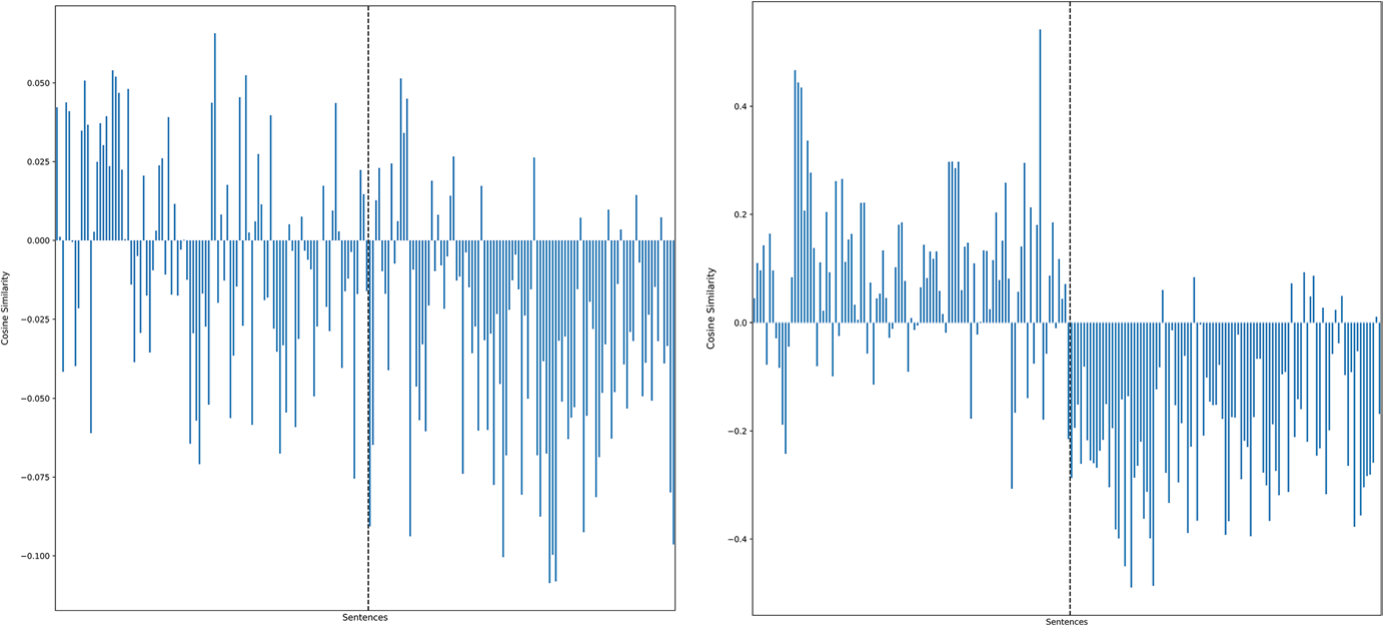
A visual comparison of using the vector ${\overrightarrow{v}}^{f}$ for *‘it was fair – it was unfair’* (left panel) vs. the fairness perceptions vector $\overrightarrow{v}$ (right panel) with a list of fair and unfair sentences. Sentences to the left of the dotted line in each panel ought to be positive, while those to the right of the dotted line in each panel ought to be negative. Higher accuracy is found for the fairness perceptions vector $\overrightarrow{v}$ with almost all unfair acts correctly classified as detailed in the confusion matrix seen in Table 3

The above examples use 18 fair and 18 unfair illustrative sentences. For a more rigorous test, we used the fairness vector $\overrightarrow{v}$ with the full list of 200 sentences (Appendix 2), which we find produces an F1 = 79.8, Precision = 88.0, Recall = 73.0, and Accuracy = 81.5. This may be compared to an F1 = 55.2, Precision = 45.0, Recall = 71.4, and Accuracy = 63.5, found when using the vector ${\overrightarrow{v}}^{f}= \overrightarrow{^{\prime\prime}it \ was \ fair^{\prime\prime}}- \overrightarrow{^{\prime\prime}it \ was \ unfair^{\prime\prime}},$ as given in the confusion matrix in Table 3 and 4 respectively. The numbers within each table refer to the number of sentences in each class. This is shown diagrammatically in Fig. 4.

**Table 3:** Confusion matrix for testing the fairness vector $\overrightarrow{v}$ against the full list of sentences

*N* = 200		Vector used $\overrightarrow{v}$	
		Actual Class	
		Fair	Unfair
Predicted Class	Fair	73%	10%
	Unfair	27%	90%

**Table 4:** Confusion matrix for testing vector ${\overrightarrow{v}}^{f}$against the full list of sentences

*N* = 200		Vector used ${\overrightarrow{v}}^{f}$	
		Actual Class	
		Fair	Unfair
Predicted Class	Fair	45%	18%
	Unfair	55%	82%

Given we used the USE to encode the test sentences and fairness vector, we repeat the experiment using SBERT. This produces an F1 = 96.9, Precision = 99.0, Recall = 95.0, and Accuracy = 97.0, with the confusion matrix reported in Table 5. The improved results obtained for SBERT are expected given its efficient solution to producing sentence embeddings by using a siamese network architecture as detailed above.

**Table 5:** Confusion matrix for testing the fairness vector $\overrightarrow{v}$ against the full list of sentences using SBERT

*N* = 200		Vector used $\overrightarrow{v}$	
		Actual Class	
		Fair	Unfair
Predicted Class	Fair	95%	91%
	Unfair	5%	99%

#### Performing a sentiment analysis on the sentences

A correlation of sentiment score using Vader (Hutto, 2021) and the fairness vector’s score for all 200 sentences encoded using the USE is then carried out thereafter and found to be 0.66, indicating that the fairness vector based on psychological factors $\overrightarrow{v}$, captures dimensions beyond that of sentiment. For illustrative purposes, we compare the sentiment scores for the sentences below (Table 6) with the result found in using the fairness vector.

**Table 6:** Comparison of a number of uncorrelated results found when performing sentiment analysis on a list of illustrative sentences against the use of the parsimonious representation of fairness given in vector $\overrightarrow{v}$

Sentence	Negative	Neutral	Positive	Compound	Sentiment Analyser Outcome	Fairness Perceptions Vector $\overrightarrow{v}$	Fairness Vector Outcome
The jury convicted the innocent	0.000	0.625	0.375	0.3400	Incorrect	-0.168450	Correct
The army executed the innocent	0.000	0.625	0.375	0.3400	Incorrect	-0.232097	Correct
The man scratched the baby	0.000	1.000	0.000	0.000	Incorrect	-0.150248	Correct
the manager helped the bullied	0.506	0.494	0.000	-0.6249	Incorrect	0.131304	Correct

The sentiment outcome for each sentence is incorrect when considering whether or not it reflects a fairness sentiment – where a positive outcome ought to reflect a fair sentence

We repeat this using a pre-trained RoBERTa language model based sentiment analyser (Barbieri et al., 2020) for which we obtain a correlation score of 0.62. For illustration purposes we compare the sentiment scores found using this method with those found using the fairness vector for the same sentences (Table 7).

**Table 7:** Comparison of a number of results found when performing sentiment analysis using RoBERTa on a list of illustrative sentences against the use of the parsimonious representation of fairness given in vector $\overrightarrow{v}$

Sentence	Negative	Neutral	Positive	Sentiment Analyser Outcome	Fairness Perceptions Vector$\overrightarrow{v}$	Fairness Vector Outcome
The jury convicted the innocent	0.188	0.713	0.099	Incorrect	-0.168450	Correct
The army executed the innocent	0.878	0.113	0.009	Correct	-0.232097	Correct
The man scratched the baby	0.495	0.475	0.030	Correct	-0.150248	Correct
the manager helped the bullied	0.472	0.505	0.022	Incorrect	0.131304	Correct

The sentiment outcome for each sentence is incorrect when considering whether or not it reflects a fairness sentiment – where a positive outcome ought to reflect a fair sentence

Such a result is not surprising, as a fairness perception vector represents dimensions beyond those of positive and negative affect – although some overlap is expected, given that positive sentiment is typically associated with fair outcomes.

#### Results from approach 2

**Fig. 5:**
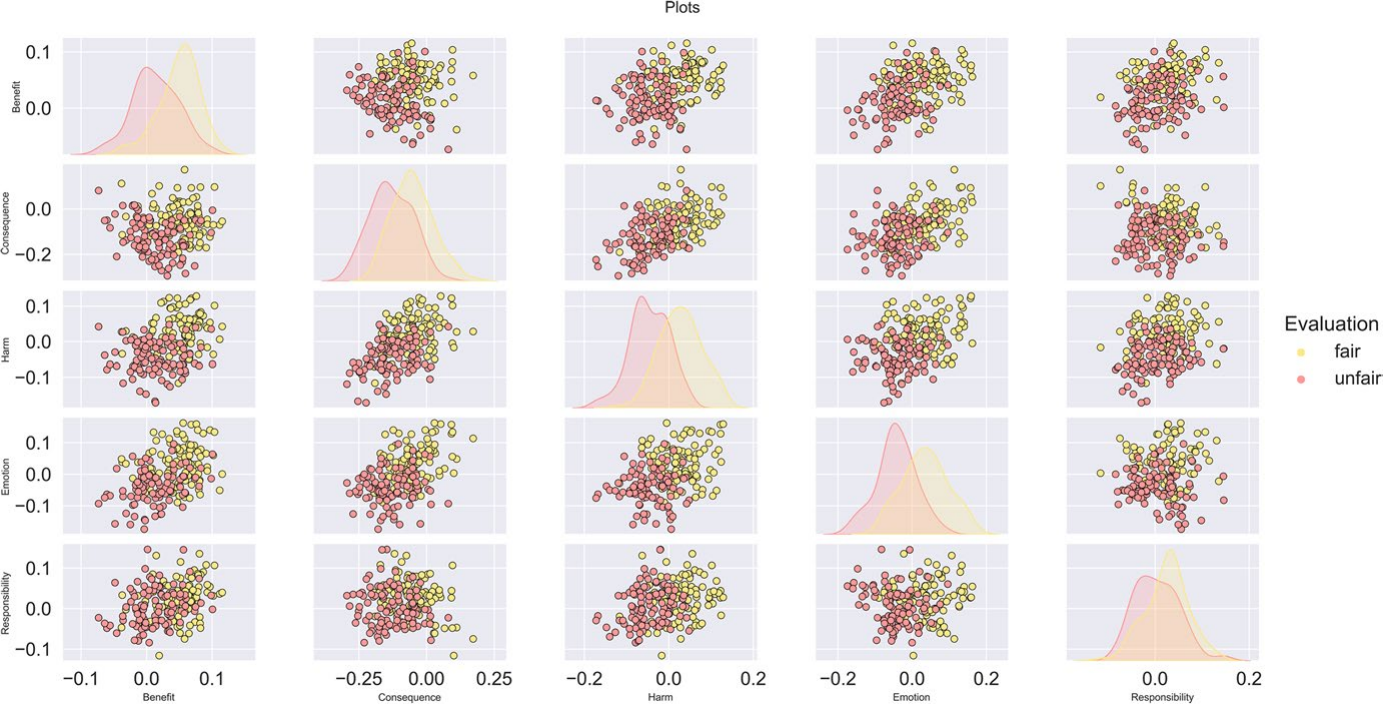
All dimensions using the vector ${\overrightarrow{v}}^{m}$ plot against each other using a scatter plot for results found using the USE

The dataset D1 is built, containing the vector ${\overrightarrow{v}}^{m}.$Whereby the result of each vector comparison is stored in a single matrix, e.g., ${\overrightarrow{v}}^{m}=\left[\text{0.2,0.1,0.6,0.3,0.2}\right]$. Each assessment is hand labelled as fair or unfair. The scatter plot for the dimensions can be seen below in Fig. 5.

**Fig. 6:**
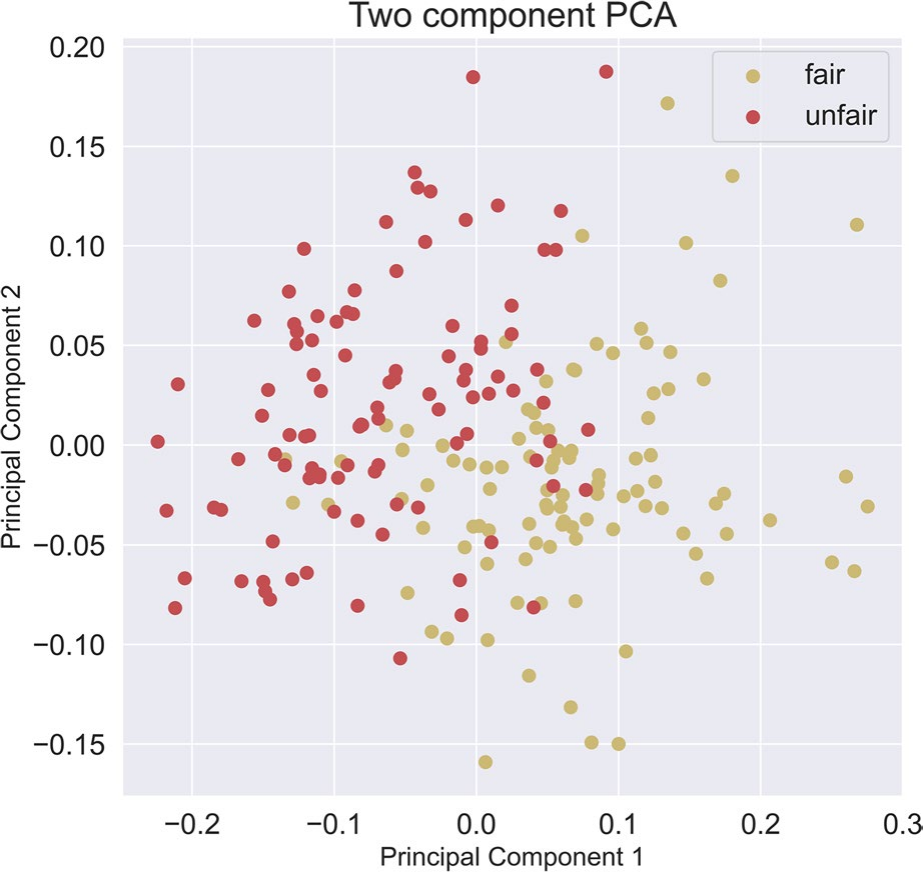
PCA on the data set, 74% explained in first two components. The explained variance ratio for the PCA is found to be 0.56, 0.18, 0.15, 0.08

Performing a two component PCA on the dataset D1, Fig. 6:

Using a PCA, set for 95% of the variance, we then perform the ML step using a logistic regression classifier, with a test split of 1:7. The result is an F1 = 86.2, Accuracy = 86.0, Precision = 88.0, Recall = 88.0.

**Fig. 7:**
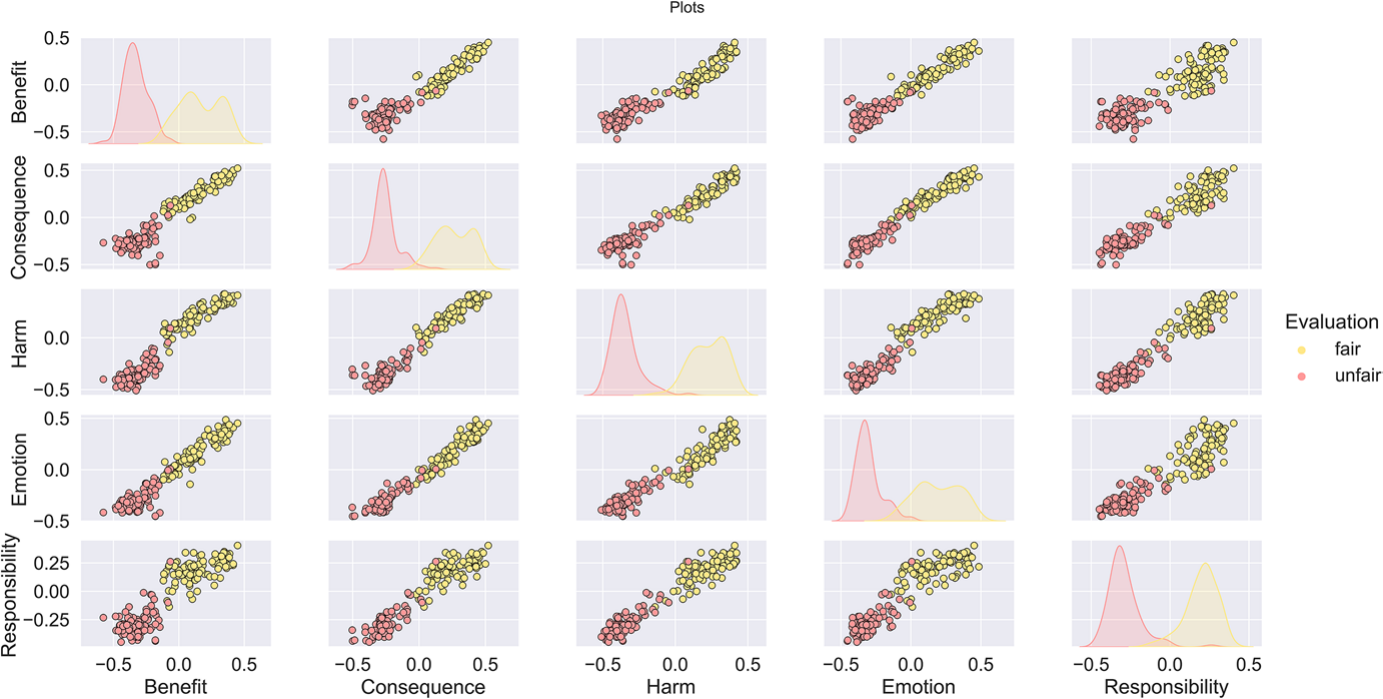
All dimensions using the vector ${\overrightarrow{v}}^{m}$ plot against each other using a scatter plot for result using SBERT

**Fig. 8:**
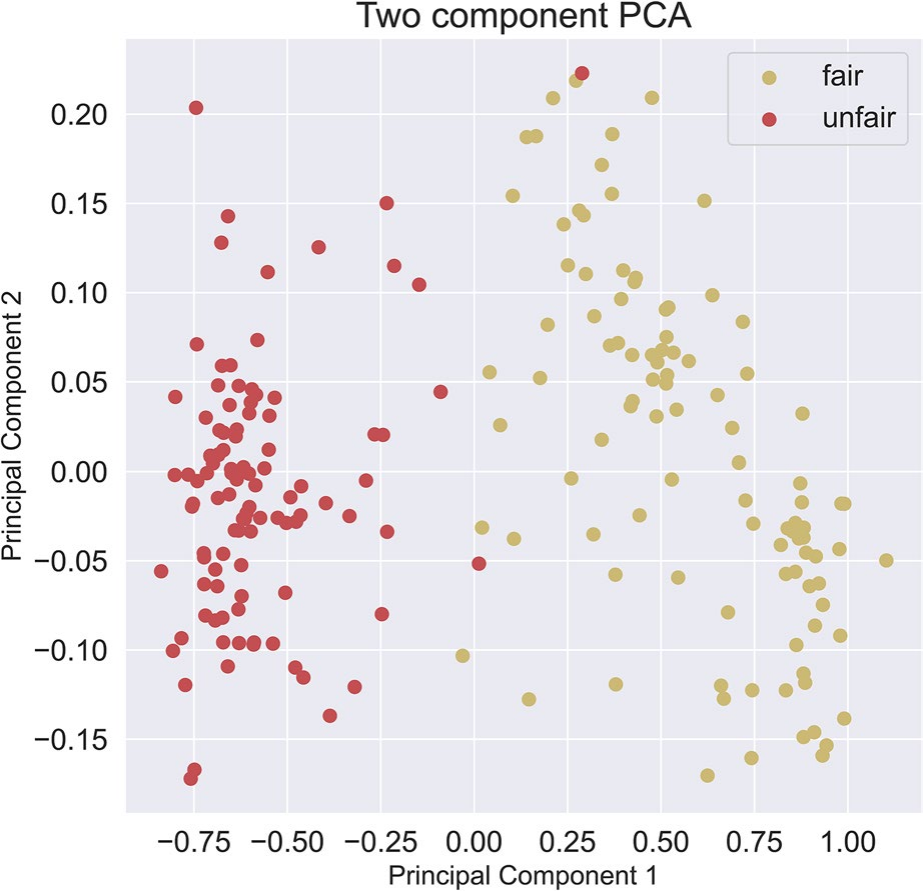
PCA on the dataset found using SBERT, 99% explained in first two components. The explained variance ratio for the PCA is found to be 0.97, 0.02

Repeating the tests using SBERT instead of the USE produced a scatter plot for the dimensions as given in Fig. 7, and PCA plot of Fig. 8.

Using a PCA, set for 95% of the variance, we then perform the ML step using a logistic regression classifier, with a test split of 1:7. The result is an F1 = 100.0, Accuracy = 100.0, Precision = 100.0, Recall = 100.0.

## Discussion

In order for a vector to approximate how *fair* or *unfair* a sentence is, the terms used in the vector must reflect the social ontological properties of fairness. That is, abstractions which make them more likely to be used and hence co-occur within a corpus with fair or unfair terms. While social rules and conventions differ between societies, the paper sought to leverage a higher abstraction of those social rules, abstractions which we *induced* from the psychology literature.

Humans prefer to be on the receiving end of pro-social than anti-social acts, irrespective of culture. This natural human bias is held within corpora (Jentzsch et al., 2019; Schramowski et al., 2019; Izzidien, 2022) that contain typical human textual discourse – i.e., not corpora built only on fantasy novels, where human acts are morphed for dramatic effect, such as descriptions of societies where eating elderly people is a norm.

As such, it may be possible to leverage this bias to act as a metric. Typically metrics are based on predefined conventions, which are reached through agreement, e.g., the length of a centimetre, or through *deduction* from survey data, such as the Big Five personality test (Raad & Perugini, 2002). We have argued that it is possible to leverage the uniqueness of human language within vector space, without the need to arrange for agreement on a fairness list or template of Do’s and Don’ts. For the first approach used in this paper (approach 1) there was no need for ML training, which is atypical for a classification task. Instead, we assembled a new vector to represent fairness perceptions.

In using human readable terms for the vectors, the outcome has a degree of explainability, which has been seen as necessary for more ethical AI (Mathews, 2019) as well as offering a conduit to audit the pipeline of the metric (Mökander & Floridi, 2021).

In the second approach we used the results to train a ML algorithm. The latter approach improved classification (F1 = 86.2) compared to the former (F1 = 79.8). However, one advantage of the former approach is that, as mentioned, it offers an added explainability of its results. Since the classification of a sentence is based on known variables which can be displayed to a user. Although the ML approach does improve on these results, being based on more data points, a degree of explainability is lost in using the logistic regression classification. However, it would be of interest to use more modern ML algorithms, such as those that use deep learning, as well as more recent sentiment analysers for further analysis. Modern sentence embedding methods based on transformer language models might bring significant improvements while also providing explanatory power. For instance Generative Pre-trained Transformer (GPT3) or Pathways Language Model (PaLM) based models can explain their reasoning in natural language.

It may be argued, that while ML was used in the second approach, it does offer a degree of explainability over other approaches that directly vectorise test sentences and incorporate training labels leaving the ML algorithm open to ‘choose’ which of the many social dimensions held in language will be used to make the classification.

While we used the psychology literature to find the principal factors that explain fair acts, it may be argued that the list of terms used is not exhaustive. Indeed, other factors do come into play, for example, ‘a feeling of guilt’ (Cartwright, 2019). However, these factors are typically contingent on the principal factors outlined in the paper, i.e., a feeling of guilt cannot manifest if there has been no perception of the possible harm and loss. Or it was the case that these additional factors were shown to have less explainability of the variance in the social psychology literature (Engel, 2011). Yet. It is still possible to add these as additional vectors to improve the measure.

Ideally, the wording of the terms used ought to be derived from the corpus itself instead of using human input as we have done. This is based on the premise that a social bias exists within the corpus, and that through an automated selective sampling of terms using a feedback loss mechanism, the most explanatory terms may be found for this bias, from within the corpus.

A number of limitations of the measure, as it stands, are detailed next.

## Limitations and further work

The above vectors in $\overrightarrow{v}$ are not fully linearly independent due to conceptual overlaps between the terms mentioned in each vector. Indeed, achieving full linear independence in measures that have a psychological dimension may not be fully achievable. Yet, an alternative approach could be to use sub-space projections. Thus, if instead of summing the vectors, we can use them to form a basis for a subspace. We can then represent any other sentence vector in the ambient embedding space by its projection onto that subspace.

If we were to define the subspace as ℂ, the vectors can be used as a basis $B$ = $\left\{{\overrightarrow{v}}^{\left(1\right)},{\overrightarrow{v}}^{\left(2\right)},{\overrightarrow{v}}^{\left(3\right)},{\overrightarrow{v}}^{\left(4\right)},{\overrightarrow{v}}^{\left(5\right)}\right\}$ for ℂ. Here any vector in the subspace will be a linear combination of the form:

4 $$\overrightarrow{v}=\alpha {\overrightarrow{v}}^{\left(1\right)}+\beta {\overrightarrow{v}}^{\left(2\right)}+\gamma {\overrightarrow{v}}^{\left(3\right)}+\delta {\overrightarrow{v}}^{\left(4\right)}+\varepsilon {\overrightarrow{v}}^{\left(5\right)}$$

Thus, instead of simply taking dot products with these vectors, a projection of any sentence in our model onto ℂ, which is defined to be the linear span of *B*, will be possible. For example, a vectorised test sentence $\overrightarrow{t}$ can be represented as below, with $\overrightarrow{o}$ being a factor that resides in the orthogonal complement of the subspace

5 $$\overrightarrow{t}=\alpha {\overrightarrow{v}}^{\left(1\right)}+\beta {\overrightarrow{v}}^{\left(2\right)}+\gamma {\overrightarrow{v}}^{\left(3\right)}+\delta {\overrightarrow{v}}^{\left(4\right)}+\varepsilon {\overrightarrow{v}}^{\left(5\right)}+\overrightarrow{o}$$

A computation to find the coefficients being possible by taking inner products of each basis vector of ℂ with both sides of the above equation for $\overrightarrow{t}$. Subsequently, we can perform a PCA, finding a separating hyperplane with the highest margin, or perform any unsupervised clustering scheme, in order to produce the two clusters representing fair vs. unfair projections.

**Fig. 9:**
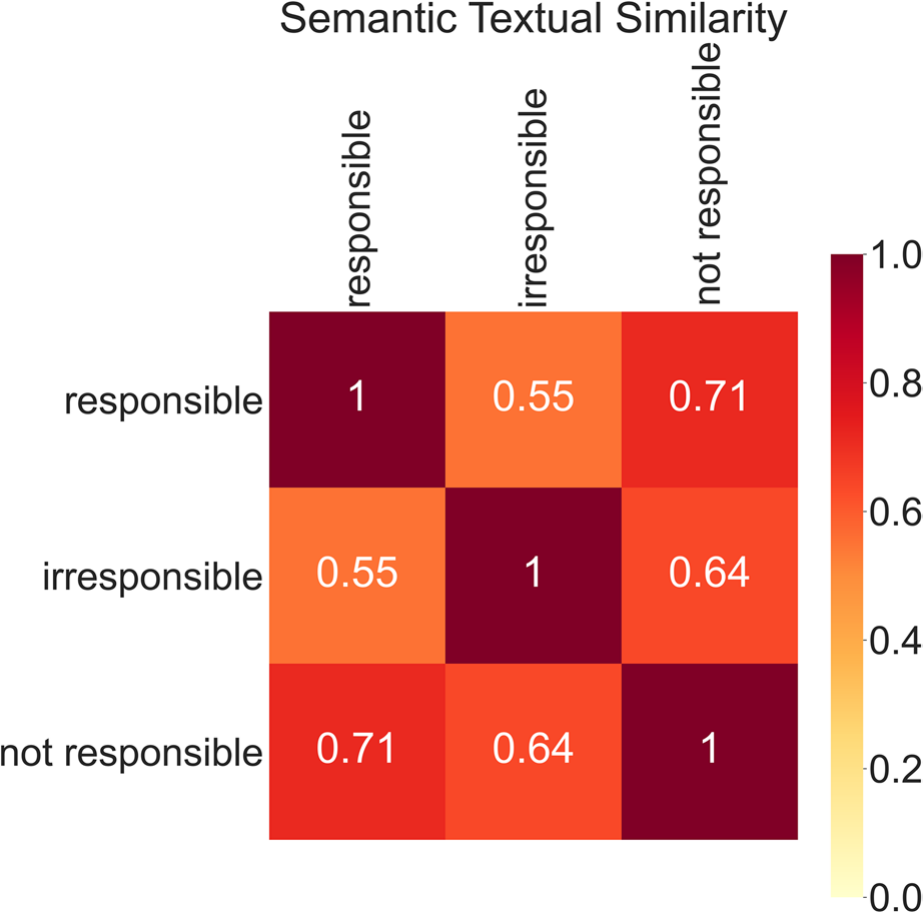
Heat map displaying similarity scores for permutations of the term ‘responsible’ in the vector embedding

A further limitation comes in using language models. While they can be used to embed texts, comparing them is not necessarily one of comparing meaning. For example, we tested this on the USE by plotting a heatmap of similarities between the word ‘responsible’, ‘irresponsible’, and ‘not responsible’. Despite a similarity in meaning, the similarity scores found using cosine similarity were different. The opposite sense of ‘responsible’ i.e., ‘irresponsible’ was more dissimilar than ‘not responsible’, (Scores: 0.55 vs. 0.71), Fig. 9 below.

A further limitation lies in the problem of pieces of anti-social biased texts within the corpora. If it were deemed *responsible* to hurt someone because of their skin colour, for example, this bias may find its way into the fairness perception metric. In this circumstance, the use *responsible* in such a singular context would refer to a negative act. To address this, we propose a further metric to be used in conjunction with a *fairspace* subspace projection. Namely, the use of the Golden Rule (GR), that is, to do onto others as one would have them do unto oneself (Izzidien & Stillwell, 2021). Whereby a fair act is one that one would be accepting of if it were done onto oneself. Using the logic of the GR, we can assume that no one wishes to be hurt because of their skin colour. Thus, in re-formulating the fairness perception metric to incorporate such a heuristic, it may be possible to avoid such pitfalls. Even if the corpora contain instances of praise for such anti-social acts, reformulating them by asking if the perpetrator would wish this upon themselves offers a possible avenue out of this bias. Once this limitation is addressed, a repeat of the whole process using subspace projections, and a list of thousands of sentences should be completed, in further work on the topic.

### Concluding remarks

Some argue that fairness has origins in human nature, with others pointing to social constructivism (Brewer, 2004; Corradi-Dell’Acqua et al., 2013). In either case, its representation in language appears to offer a feature that can be used to capture the dimensions of a fairness perception. We hypothesised that *fairness*, being a collection of social ontologies, may be partly represented using vectors. We used this assumption to ‘triangulate’ the term, i.e., *fairness*, by outlying its main ontologies based on findings from the psychology literature. In using representative corpora of human language, it was argued that it is possible to class sentences using this ‘triangulation’ as being closer to being perceived as fair or unfair, by leveraging an inherent bias. That this bias has its roots in humans being a social species that prefers fair outcomes over unfair ones. This paper represents its use in the specialised domain of measuring fairness perceptions, as applied to the digital humanities. A number of further steps must still be taken to produce a fairness metric, such as the digitisation of the golden rule, whereby fair acts are classified as those that an individual would be willing to receive. As well as the implementation of subspace projections for orthonormal representations of the vectors that represent perceptions of fairness.

## Data Availability

Data sharing not applicable to this article as no datasets were generated or analysed during the current study.

## Appendix 1

Table 8

**Table 8:** List of 18 fair and 18 unfair sentences selected at random from the longer 200 list. The selection serves for illustrative purposes on vector addition and subtraction outcomes in the paper

Fair	Unfair
The baby smiled at the father	The man harmed the lady
The man excused the visitor	The footballer damaged the goalkeeper
The lecturer amused the students	The teenager slandered the attendant
The woman picnicked with the man	The student slurred the teacher
The nurse snuggled the baby	The killer disfigured the person
Jim hugged Sara	The saboteur contaminated the people
The workers savoured the food	The guard dehumanized the boy
The man serenaded his fiancé	The spy poisoned the innocent
The groom serenaded the bride	The father murdered the boy
The president welcomed the immigrant	The attendant misinformed the customer
The student appreciated the tutor’s help	The man demonized the people
The crowd acclaimed the singer	The woman assaulted the baby
The audience enjoyed the tenor	The man sickened the lady
The principal thanked the student	The organizer mismanaged the crowd
Jack celebrated with Jill	Richard brutalized Noah
The teacher loved the pupils	The escapee raped the policeman
The nanny comforted the child	The army slaughtered the children
Tom charmed the woman	The teacher crippled the student

## Appendix 2

**Table:**

Fair	Unfair
The baby loved the mother	Jane bullied Paul
The baby loved the father	Peter killed Joe
The brother helped the sister	The man killed the man
The boy loved the girl	Tom hit Mary
The boy cradled the baby	The wife attacked the husband
The father loved the baby	Tom cut Mary
Tom liked Tim	Paul hurt Bella
Jane adored Mary	Susan killed Joe
The girl adored the actor	The boy abused the baby
The actor hugged the actress	The boy abused his sister
The actor kissed the actress	The girl blackmailed the boy
Mary adored Tim	the girl slapped the boy
The girl adored Tom	The man scratched the baby
The man thanked the man	The girl slapped the baby
The man thanked the woman	John tortured Tim
The woman thanked the man	Sally threatened Louise
The woman thanked the police	The pervert harassed the woman
The woman thanked the woman	The robber overpowered the resident
The police thanked the woman	the pervert harassed the baby
The police thanked the police	The man intimidated the girl
The husband comforted his wife	The boy harmed the baby
The groom complemented the bride	The boy mutilated the baby
Mary loved the baby	The boy poisoned the baby
The wife loved the son	The boy dismembered the baby
The man serenaded his fiancé	The boy offended the baby
Mary appreciated Mike	The boy killed the baby
The pastor thanked the priest	The boy murdered the baby
The child assisted his father	The boy hurt the baby
The man charmed the lady	The boy cut the baby
The headmistress embraced the girl	The man assaulted the lady
The tailor admired the woman	The man dehumanized the lady
The president greeted the immigrant	David killed Michael
The man loved his girlfriend	The grandfather attacked the grandchild
The police reciprocated the hero	The general killed his people
The woman admired the captain	The solider disfigured his captain
The detective welcomed the defendant	The man murdered his wife
The child cleaned the baby	The son killed the father
The sailor guided the seafarer	The bride gouged the groom
The solicitor advised the client	The baby traumatized Mary
The student tutored the pupil	The guard tortured the prisoner
The Russians helped the Americans	The colonel executed the child
The Americans helped the Russians	The interrogator burned the suspect
The student tutored the friend	The lawyer bribed the judge
The judge freed the prisoner	The man destroyed the shop
The allies freed the prisoners	The director killed the employee
The gentleman welcomed the stranger	The president rejected the refugee
The man excused the visitor	Richard killed Noah
The suitor paid the saleswoman	Richard murdered Noah
The Germans paid the Soviets	Richard terrorized Noah
The soldier saved the prisoners	Richard strangled Noah
The lady bathed the baby	The criminal tortured the victim
The child obeyed his mother	The criminal burned the victims
The waitress served the party	The thief stabbed the shopkeeper
The musician entertained the audience	The man stabbed the pedestrian
The student called the professor	Richard brutalized Noah
The man respected the professor	Joseph violated Joseph
the man hired the workman	Patricia assaulted David
the woman hired the tailor	The burglar threatened the homeowner
the manager helped the bullied	Rebecca neglected the baby
The husband dined the wife	Jonathan tortured the kid
Mary taught Sam	The man rejected the lady
The husband hugged the wife	The lady rejected the man
The driver found the party	Susan abused Kim
The minister loved the congregation	Susan insulted Timothy
The girl appreciated the suitor	The child violated the child
The athlete cheered the crowd	The man raped Patrick
The man adored his wife	The mother murdered Henry
The driver delivered the passengers	The female killed the male
The driver comforted the passengers	The party insulted the guest
The actor romanced the actress	The guest disfigured the lady
The headmaster amazed the pupil	James betrayed John
The headteacher taught the pupils	The manager extorted the employee
The president obeyed the senate	Jenifer blackmailed the boyfriend
The worker praised the workmen	Jenifer assassinated the gardener
The worker raised the workmen	The horticulturist poisoned the pensioner
The lady beautified the girlfriend	The government terrorized the people
The security trusted the manager	The state murdered the prosecutor
The manager energized the employee	The army deposed the winner
The singer excited the audience	The crowd mobbed the prosecutor
The singer enthused the boy	The crowd killed the protestor
The pilot charmed the stewardess	The army executed the innocent
The teacher loved the pupils	The caretaker poisoned the household
The actor heroized the protagonist	The mother decapitated the child
The doctor treated the patient	The gang burnt the lion
The farmer nourished the child	The corporation polluted the ocean
The farmer fostered the family	The locksmith robbed the landlord
The caretaker cleaned the house	The university silenced the professor
The nurse cleaned the patient	The university housed the students
The scientist taught the attendee	The professor cheated the students
The boy hugged the uncle	the attacker slashed a stranger
The crowd cheered the singer	the thief gouged his eyes
The people loved the leader	the criminal wounded the police
The nurse treated the patient	usher scolded the protestors
The surgeon admitted the patient	protestors hit the police
The lecturer amused the students	protestors kicked the police
The researcher taught the class	rioters stabbed the police
The presenter surprised the audience	The rioters attacked the bystanders
The soldier saluted the general	The man killed his friend
The painter painted the woman	The clerk murdered his manager
The child praised a teacher	The jury convicted the innocent

## Code availability

https://github.com/AhmedIzzidien/Sentence-Fairness-Measure
